# A Study on the Clinical and Hormonal Profile of Polycystic Ovarian Syndrome Patients attending a Tertiary Care Hospital: A Descriptive Cross-sectional Study

**DOI:** 10.31729/jnma.5694

**Published:** 2020-11-30

**Authors:** Achala Vaidya, Sweta Yadav, Anshu Vaidya

**Affiliations:** 1 Department of Obstetrics and Gynaecology, Norvic International Hospital, Kathmandu, Nepal

**Keywords:** *body mass index*, *Nepal*, *obesity*, *oligomenorrhea*, *polycystic ovarian syndrome*

## Abstract

**Introduction:**

Polycystic ovarian syndrome is the most common endocrinological disorder in women of reproductive age and has a considerable metabolic, reproductive, and cardiovascular consequences. This study was designed to provide an overview of the clinical profile and hormonal presentation of the patients with polycystic ovarian syndrome attending a tertiary care hospital.

**Methods:**

A descriptive cross-sectional study was conducted between September 14, 2019 to October 16, 2019 on patients presenting to a tertiary care hospital, after obtaining ethical clearance from Institutional Review Committee (Dated 03/09/2019) and informed consent from the patient or patient relatives. Data entry and analysis were done in Microsoft Excel 10. The data was statistically analysed using Statistical Package for the Social Sciences version 20.0.

**Results:**

Out of 100 patients, the mean age of the patients was 24.9 ± 4.52 years, and the most common group was 26-34 years. The most common presenting symptom was menstrual irregularity which was seen in 86 (86%) of the patients, followed by weight gain in 55 (55%) of the patients. Thirty (30%) patients were overweight, while 11 (11%) of the patients had grade I obesity. LH/FSH ratio was more than or equal to 2 in eighty-three percent 83 (83%) of the patients.

**Conclusions:**

Polycystic ovarian syndrome has varying clinical manifestations, most commonly affecting the young women of reproductive age group. The commonest presenting complaint in our study was menstrual abnormality. Majority of the patients had a deranged hormonal profile which can increase the risk of cardiovascular diseases and type two diabetes. Thus, awareness regarding the disease is important for early diagnosis prevention of complications.

## INTRODUCTION

PCOS is one of the most common endocrine disorder of reproductive age affecting 5% to 10% of women worldwide.^1^ It is a heterogeneous disorder of uncertain etiology, but there is strong evidence that complex interactions between genetic, environmental, and behavioural factors contribute to causing this syndrome.^2^ The diagnosis of PCOS is made if two of the three criteria of androgen excess, ovulatory dysfunction or polycystic ovaries are met.^3^ PCOS symptoms include amenorrhea or oligo amenorrhoea, hirsutism, obesity, acne, androgenic alopecia and reproductive disorders.^4^

Patients with PCOS are at risk for a group of metabolic disorders including insulin resistance, glucose intolerance/impairment, diabetes, hypertension, lipid disorders, cardivascular disease, and increased risk of endometrial, uterine, and breast cancers.^5^ PCOS is an emerging health problem of adolescence requiring healthy lifestyles and early interventions to prevent future morbidities. Literature reviews have demonstrated that 30-70% of PCOS females are obese. Although, the disease is also seen in females of normal weight but with less frequency.^4^

This study aims to provide an overview of the presentation of PCOS in the Nepalese population attending a tertiary care hospital, specifically the clinical presentation and hormonal profile.

## METHODS

This descriptive cross-sectional study was carried out in the gynaecology out-patient department of Norvic International Hospital from September 14 to October 16, 2019. Ethical clearance was taken from the institutional review board for the study (IRC No: Dated 03/09/2019). PCOS patients were selected based on the Rotterdam's 2003 criteria.^3^ For diagnosis two of the following three criteria was required: Oligo- or anovulation, Clinical and/ or biochemical signs of hyperandrogenism, Polycystic ovaries and exclusion of other etiologies (congenital adrenal hyperplasia, androgen-secreting tumors, Cushing's syndrome).

The sample size was collected using the formula,

$$\begin{array}{ll} \text{n} & \text{= }({\text{Z}}^{\text{2}}\times \text{p}\times (\text{1}-\text{p))}/{\text{e}}^{\text{2}}\\ & \text{= }(1.96^{\text{2}}\times 0.07\times 0.93)/\text{0}{\text{.05}}^{\text{2}}\\ & \text{= }100.0\sim 100 \end{array}$$

Where,

Z = 1.96 (At 95% confidence interval)
p = prevalence, 7%
e = margin of error, 5%

Convenience sampling technique was applied. All patients who met Rotterdam's 2003 criteria for PCOS and gave consent were included, while patients not giving consent and patients with symptomatic liver, kidney, heart disease, Cushing's syndrome, adrenal hyperplasia/tumour, ovarian tumour, and pregnancy were excluded from the study.

Informed consent was taken and pre-structured proforma were filled. A detailed history was obtained. Relevant details of each participant such as age, sex, and clinical features of disease were noted. Height and weight were measured and body mass index (BMI) was calculated. World Health Organisation (WHO) and National Institute of Health (NIH) criteria was used to classify BMI<18.5 as underweight, BMI between 18.5 and 24.9 as normal weight, BMI between 25 and 29.9 as overweight and obesity as a BMI of 30 or greater. Obesity is further divided into class I (BMI 30-34.9), class II (BMI 35-39.9) and class III (BMI>40).^6^

Blood samples were taken for FSH, LH, prolactin, testosterone, thyroid stimulating hormone (TSH) and fasting serum insulin. Data entry and analysis were done in Microsoft Excel 10. The data was statistically analysed using Statistical Package for the Social Sciences (SPSS) version 20.0. Frequency, percentages was calculated for binary data.

## RESULTS

**Figure 1:**
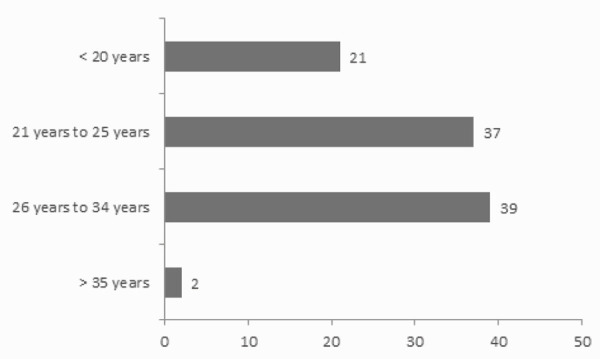
Age Distribution.

Mean age of the studied patients was as 24.9±4.52 years and the most common age group was 26-34 years in 38 (38%) (Figure 1).

**Figure 2:**
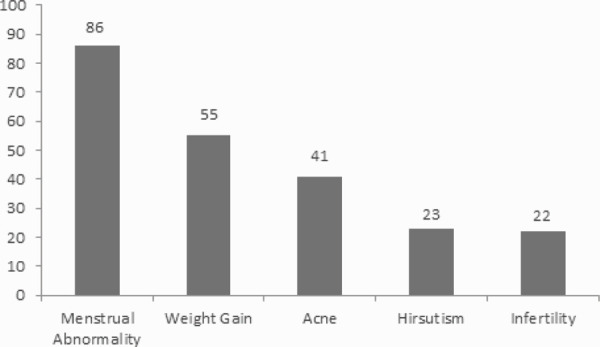
Presenting complaints.

The most common presenting symptom was menstrual irregularity which was seen in 86 (86%) of the patients, followed by weight gain 55 (55%) (Figure 2).

**Figure 3:**
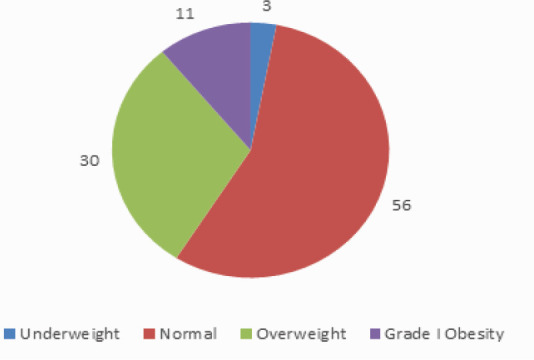
BMI of patients.

Although majority of the patients, approximately 56 (56 %) had normal BMI, 30 (30%) were overweight and 11 (11%) were obese (Figure 3).

**Figure 4:**
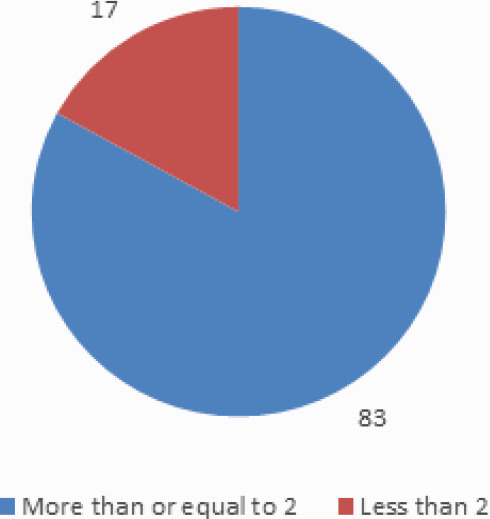
LH/FSH ratio.

Eighty three (83%) of the patients had LH/FSH ratio more than or equal to 2 (Figure 4).

Similarly, hypothyroidism was detected in twenty-one percent 21 (21%) of the patients whereas fasting serum insulin was found to be raised in thirty-three percent 33 (33%) of the total subjects (Table 1).

**Table 1:** Values of Hormones.

Hormone Level	Frequency (n)
Prolactin	
< 127	7
127 to 637	87
> 637	6
TSH	
< 0.2	2
0.2 to 4.2	77
> 4.2	21
Free Testosterone	
< 0.1	0
0.1 to 6.3	92
> 6.3	8
Fasting serum Insulin	
< 3	2
3 to 25	65
> 25	33

## DISCUSSION

PCOS is probably the most prevalent endocrinological disorder affecting females and is the most common cause of menstrual disturbance during the reproductive age. It is characterised by the presence of polycystic ovaries on ultrasound and/or clinical and biochemical signs of hyperandrogenism and/or oli-anovulation.^7^

In this study, mean age of the studied patients was as 24.9±4.52 years and the most common age group was 26-34 years (38%). This is comparable to the study done by Krithika et al., where mean age of PCOS patients was 27 + /-7.1 years.^8^ PCOS is reported to be more prevalent in younger ages (<35) than among older women, proposing that due to a physiological decline of the follicular cohort leading to a normalized ovarian ultrasonographic appearance with advancing age.^9^ This is consistent with our finding where the vast majority (>95%) of the patients included in the study were less than 34 years of age. Similarly, in a study from India, majority of PCOS belonged to age group between 26 and 30 years (43%).^10^

In our study, the prevalence of menstrual disorders was 86%, which is similar to the findings of the study by Joshi et al., where the prevalence of menstrual irregularity was 83%.^11^ However, a study conducted by Sunitha J Ramanand et al. reported that oligomenorrheoa was present in 65% patients.^12^

Regarding the BMI, 30% of the participants were overweight. This result is similar to what is reported by Kiddy et al., who found that 35% of the women with PCOS are overweight.^13^

Androgenic features were also common presenting complaints. Acne was observed in 41% of the patients. In a study done by Spandana JC et al.,^10^ acne was seen in 20% of the patients which is half of what we reported. Kilkenny et al. reported the incidence of moderate to severe acne to be 17%.^14^ The higher incidence of acne reported in our study maybe due to the lack of awareness about PCOS which causes delay in diagnosis and treatment of PCOS. Many women aren't diagnosed until they struggle with fertility. Prevalence of hirsutism in this study was 23%. Hirsutism incidence varied in different parts of the world from 3% in Japanese women to 70% in Caucasians women.^15,16^

In our study, infertility was reported in 22% of the patients. In a study done in Tanzania by A. B. Pembe, the prevalence of infertility was 39.4%,which is quite high as compared to our study.^17^

Disturbance in the pulsatile nature of gonadotrophin releasing hormone (GnRH) results in the relative increase in LH to FSH release.^18^ An abnormal feedback mechanism by ovarian estrogen is blamed to play role in this discriminated increase in LH release.^19^ As a result of this derangement, the ratio between FSH and LH levels which is normally around 2 to 1, become reversed and sometimes even more (2 or 3 to 1) in approximately 60% of the patients with PCOS,^20^ which correlates with the findings of our study where more than 2/3^rd^ of the women with PCOS have high LH/FSH ratio (LH/ FSH ratio >=2 in 83%).

In our study, serum prolactin level was high in 6 % of the patients, which is less than that reported in the study by Spandana JC et al.,^10^ but the prevalence of hypothyroidism (22%) in the same study was comparable to our study (21%).

This study had some limitations. This was a single centre study and sample size was small. Also, as this was a cross sectional study, long term complications and sequel of PCOS could not be studied.

## CONCLUSIONS

PCOS has varying clinical manifestation including gynecologic, endocrine and dermatologic manifestations, most commonly affecting the young women of reproductive age group and can lead to reproductive, metabolic and oncological complications in the long term. Majority of the patients present with elevated LH/ FSH ratio. Thus, PCOS should be diagnosed and treated early in adolescence. Furthermore, studies should be conducted on a larger sample size. Longitudinal studies must be carried out to determine the long term implications of PCOS in female health in Nepal.
